# Supplementary figures and images for: Vesicular Glutamate Transporter 3 Is Involved in Glutamatergic Signalling in Podocytes
Source: Int J Mol Sci. 2025 Mar 11;26(6):2485. doi: 10.3390/ijms26062485 (PMC11941860; doi:10.3390/ijms26062485)

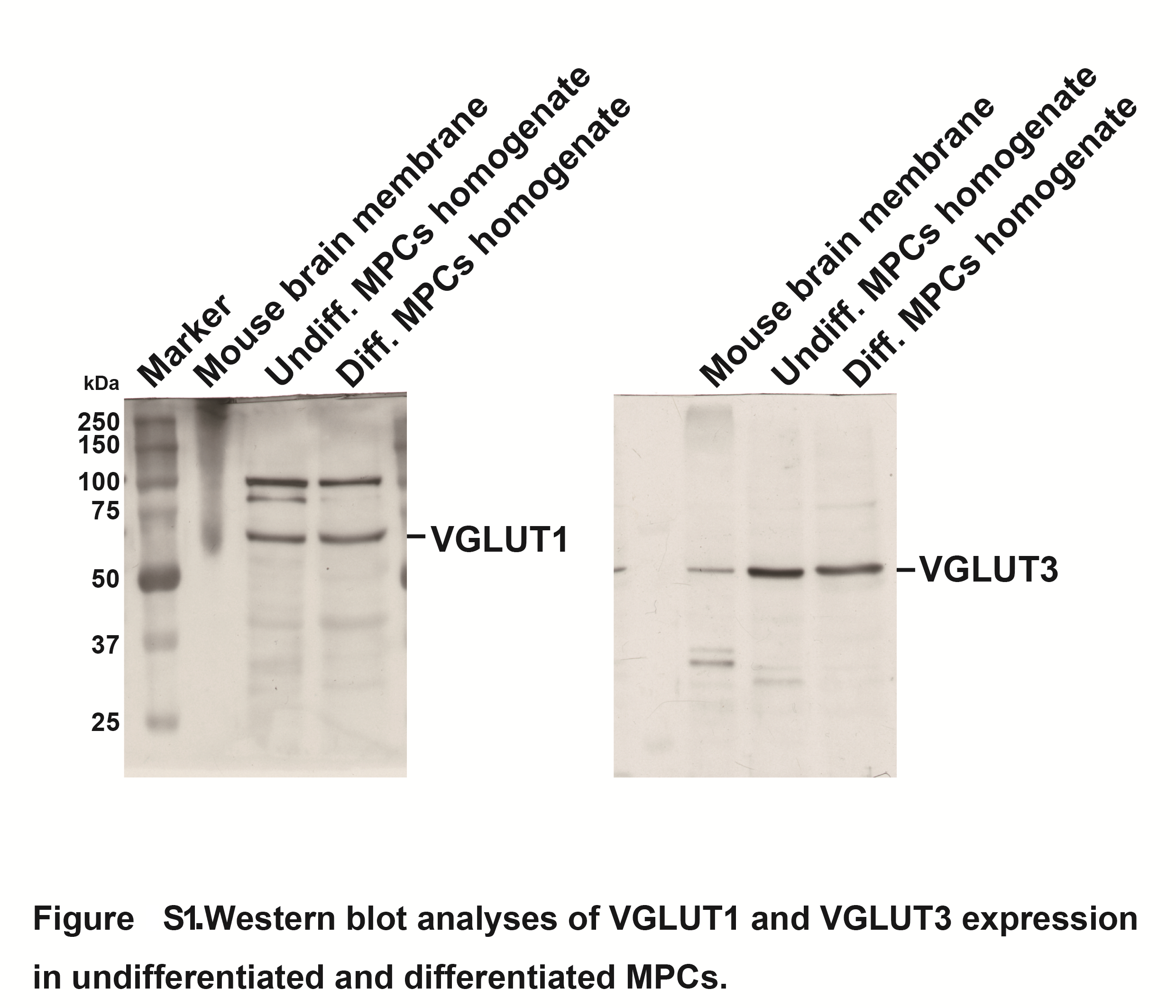

Supplement: Supplementary file 1 [file ijms-26-02485-s001.zip › Figure S1 ver 2.tif]

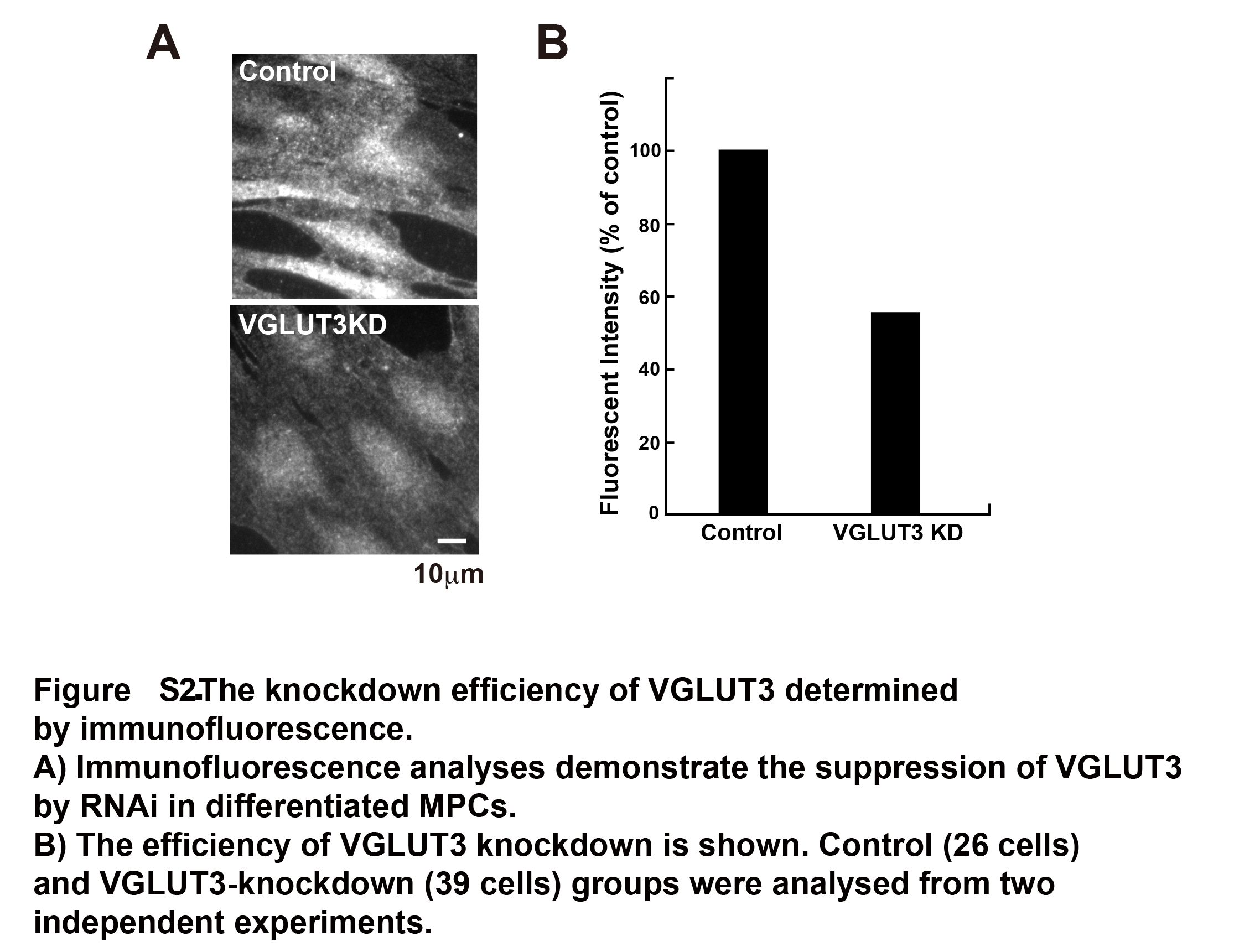

Supplement: Supplementary file 1 [file ijms-26-02485-s001.zip › Figure S2 ver 2.tif]
